# Extraction Optimization, Purification, Antioxidant Activity, and Preliminary Structural Characterization of Crude Polysaccharide from an Arctic *Chlorella* sp.

**DOI:** 10.3390/polym10030292

**Published:** 2018-03-09

**Authors:** Hong Song, Meilin He, Chuankun Gu, Dong Wei, Yuqi Liang, Junmei Yan, Changhai Wang

**Affiliations:** Jiangsu Provincial Key Laboratory of Marine Biology, College of Resources and Environmental Science, Nanjing Agricultural University, 1 Tongwei Road, Nanjing 210095, China; songhong8912@163.com (H.S.); hemeilin@njau.edu.cn (M.H.); 2016103007@njau.edu.cn (C.G.); 13260980201@163.com (D.W.); 18931330996@163.com (Y.L.); Ninglyq@163.com (J.Y.)

**Keywords:** arctic *Chlorella* sp., polysaccharide, antioxidant activity, preliminary structural characterization

## Abstract

The arctic strain of *Chlorella* sp. (*Chlorella*-Arc) exists in the coldest and driest arctic ecosystems, and it is a new resource of active polysaccharides. The extraction of crude polysaccharide from *Chlorella*-Arc was optimized using the response surface methodology. A crude polysaccharide yield of approximately 9.62 ± 0.11% dry weight was obtained under these optimized conditions. Three fractions (P-I, P-II, and P-III) were present after purification by 2-diethylaminoethanol Sepharose Fast Flow and Sephadex G-100 chromatography. The P-IIa fraction demonstrated significant antioxidant activities. Moreover, P-IIa was an α- and β-type heteropolysaccharide with a pyran group and contained variable amounts of rhamnose, arabinose, glucose, and galactose based on fourier-transform infrared spectroscopy, high-performance liquid chromatography, and ^1^H and ^13^C nuclear magnetic resonance imaging. Production of high amounts of polysaccharide may allow further exploration of the microalgae *Chlorella*-Arc as a natural antioxidant.

## 1. Introduction

Oxidation is an essential biological process for energy production in many living organisms. However, abundant reactive oxygen species are produced in vivo during some oxidative reactions. These oxygen species can react with macromolecules, leading to cellular damage [1]. Some studies have reported the health benefit of a diet rich in antioxidants, which can protect an organism against the damage caused by radicals. Natural antioxidants from seaweeds play an important role in protecting against various diseases and aging processes [2]. Moreover, antioxidant activity has been reported for polysaccharides of numerous genera of algae, including *Spirulina*, *Chlorella*, *Ahnfeltiopsis*, *Colpomenia*, *Halymenia*, *Laurencia*, and *Padina* [3,4,5]. The detected antioxidant compounds in these and other seaweeds have potential applications as immunomodulators, antitumor and anti-inflammatory agents, and antioxidants [5,6,7,8]. Over the last years, the natural sources of antioxidant extracts from seaweeds have been progressively studied and well-developed in many countries. Seaweeds polysaccharide have been considered to scavenge superoxide radicals, peroxyl radicals, nitric radicals, (1,1-diphenyl-2-picrylhydrazyl radical, DPPH) radicals, ferric reducing antioxidant power, and chelate ferrous ions. It was suggested that polysaccharide derived from marine sources such as seaweeds may have the potential to be used in the food and pharmaceutical industries [8]. The presence of sulfate groups in seaweed polysaccharides was suggested to play an important role in enhancing the antioxidant activities [9,10].

*Chlorella*, which belongs to the phylum *Chlorophyta*, is a genus of single-celled green alga. It is a source of polysaccharides, amino acids, vitamins, fatty acids, and minerals, exemplifying the high nutritional value of green algae [4,11,12]. The extraction yields and bioactivities of polysaccharides vary among the different *Chlorella* species. Hu et al. extracted polysaccharides from *Chlorella pyrenoidosa* using the supercritical carbon dioxide method through an orthogonal experiment (L16 (4^5^) and obtained 16 extracts (highest yield of approximately 7.78%)). Almost all of the extracts displayed significant anti-radical (1,1-diphenyl-2-picrylhydrazyl radical, DPPH) activities, varying from 29.67 ± 0.29% to 54.16 ± 4.49% [4]. Erick et al. reported that the extraction yield of *Chlorella pyrenoidosa* was approximately 15% by hot aqueous extraction and showed that a hot water extract of *Chlorella pyrenoidosa* was an immunomodulator [11]. The polysaccharide of *Chlorella vulgaris* displayed high immunomodulatory activity, with approximately 6.06% obtained using hot water extraction [13]. Qi et al. reported an extracted polysaccharide yield of 13.10% from *Chlorella ellipsoidea*, which showed high immunomodulatory activity [14]. Most studies of the bioactivity of polysaccharides extracted from *Chlorella* have focused on antitumor and immune activities, instead of antioxidant activity.

The arctic is the coldest and driest region on the Earth. Arctic microalgae have special physiological and biochemical characteristics [15]. These microalgae exist in arctic ecosystems that are a rich resource for the development of active substances, including polysaccharides with special application value. There have been some reports of polar microalgae and their extracellular polysaccharides. These substances protect organisms within ice floes from ice-crystal damage, buffer against pH and salinity changes, and ameliorate other chemical stresses. These substances are significant in arctic ecosystems [16]. However, little is known about intracellular polysaccharides and their biological potential. To the best of our knowledge, no publication has described the optimization of polysaccharide extraction from polar microalgae and examined their antioxidant activity. Moreover, polar algae tolerate low temperatures and are suitable for outdoor cultivation at low temperatures [17]. Therefore, an investigation of extracted polysaccharides from polar microalgae may provide basic data for the future application of large-scale cultivation of these algae at low temperatures.

This study aimed to evaluate the extraction of crude polysaccharides from the polar microalgae *Chlorella*-Arc. A response surface methodology (RSM) was proposed to optimize the effects of water dried to a microalgae powder, extraction temperature, and time on the extraction yield of crude polysaccharide. A structural examination and evaluation of antioxidant activity of *Chlorella*-Arc polysaccharides were also performed.

## 2. Materials and Methods

### 2.1. Materials and Reagents

2-diethylaminoethanol (DEAE) Sepharose Fast Flow was obtained from GE Healthcare (Beijing, China). Sephadex G-100 chromatography and standard monosaccharides (analytical grade of glucose, xylose, galactose, rhamnose, sucrose, and fructose), were purchased from Shanghai Baomanbio technology co., LTD (Shanghai, China). Dextran T-500 (ultra pure) was provided by Sigma (Shanghai, China). Bovine serum albumin (ultra pure) and coomassie brilliant blue (ultra pure) were obtained from Beijing Solarbio technology co., LTD (Beijing, China). The other analytical grade reagents: acetone, chloroform, n-butanol, NaCl, Na_2_HPO_4_, NaH_2_PO_4_, H_2_SO_4_, phenol, ethanol, DPPH radical, H_2_O_2_, pyrogallol, HCl, trifluoroacetic acid (TFA), ascorbic acid (Vc), BaCl_2_, gelatin, K_2_SO_4_, trichloroacetic acid (TCA), KNO_3_, K_2_HPO_4_, MgSO_4_·7H_2_O, Ca(NO_3_)_2_, biotin, vitamin B_12_, thiamin (B_1_), FeSO_4_·7H_2_O, Na_2_EDTA, MnCl_2_·4H_2_O, CoSO_4_·7H_2_O, CuSO4·5H_2_O, H_3_BO_3_, (NH_4_)_6_Mo_7_O_24_·4H_2_O, NiSO_4_·6H_2_O, NH_4_VO_3_ and ZnSO_4_·7H_2_O were purchased from Nanjing Sode Test Equipment Co., LTD (Nanjing, China).

### 2.2. Algae Growth Conditions

*Chlorella*-Arc cells were isolated from glacier melt water collected near China’s Yellow River Station in the Ny-Alesund area (78°55′ N; 11°56′ E, Spitsbergen Island). *Chlorella*-Arc cells were obtained from the polar research laboratory of the State Oceanic Administration of Polar Science of China. Its rbcL sequence was sent to Genebank and blasted (Genebank number: KM514914). The algae was pre-cultured in Bourelly medium [18] at 3 °C and the cells were inoculated into fresh Bourelly medium. It was grown in an Erlenmeyer flask (volume: 2 L), temperature was set to 15 °C and a light intensity of 5000 lux with a light/dark cycle of 12 h:12 h with continuous bubbling air. The algal cells were grown for 20 days to reach the early stationary phase with a cell density of 3.46 × 10^7^ cells/mL. The biomass was collected by centrifugation at 8000 rpm for 15 min, and then was vacuum freeze-dried for 24 h to obtain microalgae powder.

### 2.3. Polysaccharide Extraction

Crude polysaccharide was extracted using the hot water extraction method [19]. Before the extraction, water and dried microalgae powder were mixed, and the mixtures were treated using ultrasonic rod crasher (JY92-II, Ningbo Scientz biotechnology Co., Ltd., Ningbo, China) at 1000 W for 10 min to destroy cell walls, and this procedure was performed on ice. Three factors were optimized to obtain the maximum polysaccharide yield: the ratio of water to dried microalgae powder, extraction temperature, and extraction time. Each pretreated sample mentioned above was extracted under ratio of water to dried algae powder (volume: dry weight, mL:mg; 10:1, 20:1, 30:1, 40:1, and 50:1), extraction temperature (50, 60, 70, 80, 90 °C) and extraction time (1, 2, 3, 4, 5 h). One factor is changed, while the other factors keep constant in each experiment.

After extraction, the mixtures were centrifuged at 8000 rpm for 10 min. The supernatants were collected, and the polysaccharide precipitated by the addition of pure ethanol. The pellets were refluxed three times to remove any lipids with a mixture of acetone and chloroform (1:3, *v*/*v*). Residual proteins were removed using Sevage reagent (a 4:1 *v*/*v* mixture of chloroform and n-butanol) [20]. The protein precipitate was separated by centrifugation (4000 rpm for 15 min) and discarded, and the supernatants were collected and dialyzed in 14,000 Da dialysis bags for 48 h and precipitated by the addition of ethanol. The pellets were dried using a lyophilizer (Beijing Boyikang experimental instrument co., Ltd., Beijing, China) for 6 h to obtain crude polysaccharide powder. The crude polysaccharide yield (%) was calculated as:

(1) $$\mathrm{The}\;\mathrm{crude}\;\mathrm{polysaccharide}\;\mathrm{yield}\;\%=\frac{\mathrm{Crude}\;\mathrm{polysaccharide}\;\mathrm{weight}\;(\mathrm{mg})}{\mathrm{Dried}\;\mathrm{microalgae}\;\mathrm{powder}\;(\mathrm{mg})}\times 100\%$$

### 2.4. RSM Experimental Design

The main factors affecting polysaccharide yield (%) were the ratio of water to dried microalgae powder (mL:g), extraction temperature (°C), and extraction time (h). These three factors at their three levels are shown in Table 1.

### 2.5. Purification of Crude Chlorella-Arc Polysaccharide 

The crude polysaccharides (~2 g) were dissolved in 400 mL phosphate-buffered solution (PBS) (pH = 7.4). Three milliliter of this solution was loaded onto a chromatography column (1.2 cm × 20 cm) with DEAE Sepharose Fast Flow and sequentially eluted with PBS (pH = 7.4) and a gradient of 0–2.0 M sodium chloride at a flow rate of 1.0 mL/min. Each fraction (5 mL) of the eluate was collected. The carbohydrate content of each fraction was determined using the phenol-sulfuric acid method [21], and the protein content of fractions was determined using the Coomassie brilliant blue method [22]. The collected fractions were designated P-I, P-II, and P-III. Freeze-dried P-II fractions were dissolved in PBS (pH = 7.4). Five milliliters of the solution were loaded onto a Sephadex G-100 column (1.6 cm × 100 cm) and eluted with 0.3 M sodium chloride at a flow rate of 0.5 mL/min. Each 5-mL fraction was processed as described above.

### 2.6. DPPH Radical Scavenging Assay

DPPH radical scavenging activity was measured as previously described [23]. Briefly, 2.0 mL of DPPH solution (0.004% *w*/*v* dehydrated alcohol) was added to 2.0 mL of polysaccharides dissolved in distilled water at different concentrations (0.25, 0.5, 0.75, 1.0, 3.0, 5.0, 7.0, and 10.0 mg/mL). The mixtures were shaken and left standing for 30 min at 25 °C in the dark. The absorbance of each was measured at 517 nm. A lower absorbance of the reaction mixture indicated higher free radical scavenging activity. The ability to scavenge the DPPH radical was calculated using the following equation:

(2) $$\mathrm{DPPH}\;\mathrm{radical}\;\mathrm{scavenging}\;\mathrm{effect}\;\left(\%\right)=\left[1-\frac{A-A_{0}}{A_{1}}\right]\times 100\%$$

where A is the absorbance of the sample in DPPH at 517 nm, A_0_ is the absorbance of the sample in dehydrated alcohol at 517 nm (does not contain DPPH supplement), and A_1_ is the A_517_ of DPPH in distilled water.

### 2.7. Hydroxyl Radical Scavenging Assay 

The assay was done as previously described [24] with some modifications. Briefly, 2.0 mL of 6.0 mM FeSO_4_ and 2.0 mL of 6.0 mM ethanol salicylate were added to 2.0 mL of polysaccharide solution (0.25, 0.5, 0.75, 1.0, 3.0, 5.0, 7.0, and 10.0 mg/mL), then 2.0 mL of 6.0 mM H_2_O_2_ was added finally to start the reactions and incubated at 37 °C for 30 min. Fenton reaction produces hydroxyl radicals: Fe^2+^ + H_2_O_2_→Fe^3+^ + OH^−^ +·OH, then ·OH reacts with salicylate solution. The products could be measured at 510 nm. The scavenging effect on hydroxyl radical was calculated (%) as:

(3) $$\mathrm{Hydroxyl}\;\mathrm{radical}\;\mathrm{scavenging}\;\mathrm{effect}\;\left(\%\right)=\left[\frac{A_{1}-A}{A_{1}}\right]\times 100\%$$

where A_1_ is the absorbance of the blank and A is the final absorbance of each sample (the polysaccharides or ascorbic acid).

### 2.8. Superoxide Radical Scavenging Assay 

Superoxide radical scavenging activity was measured as previously described [20] with slight modifications. Briefly, 2.5 mL of 50 mM Tris-HCl buffer (pH 8.2) was maintained at 25 °C for 20 min, 4 mL of polysaccharide solution (0.25, 0.5, 0.75, 1.0, 3.0, 5.0, 7.0, and 10.0 mg/mL), and 0.6 mL of 25 mM pyrogallol solution was added. The mixture was incubated at 25 °C for 5 min and then 1 mL of 8.0 mM HCl solution was added to each mixture to terminate the reaction. The absorbances of the mixtures were recorded at 299 nm. The scavenging effect on superoxide radical was calculated (%) as:

(4) $$\mathrm{Superoxide}\;\mathrm{radical}\;\mathrm{scavenging}\;\mathrm{effect}\;\left(\%\right)=\left[1\text{−}\frac{A-A_{0}}{A_{1}}\right]\times 100\%$$

where A_0_ is the absorbance of the blank (Tris-HCl buffer instead of the sample), A_1_ is the final absorbance of a sample, and A is the background absorbance (Tris-HCl buffer instead of pyrogallol solution).

### 2.9. Ultraviolet-Visible (UV-Vis) and Fourier-Transform Infrared (FT-IR) Analyses 

The UV-Vis absorption spectra of samples were recorded using a UV-visible spectrophotometer (Beijing Purkinje General Instrument Co., Ltd., Beijing, China) in the spectral scan range of 200–400 nm^−1^ at room temperature.

The presence of various functional groups and compounds in the polysaccharide extracts were detected in the FTIR spectra as described previously [25]. The dried samples were ground with KBr powder and then pressed into a pellet for FT-IR using a Nicolet IR200 apparatus (Thermo Nicolet Corporation, Madison, WI, USA) measurement at a frequency range of 400–4000 cm^−1^ at a resolution of 4 cm^−1^.

### 2.10. Monosaccharide Compositional Analysis

P-IIa was hydrolyzed in 4 mol/L TFA for 2 h at 110 °C in a sealed glass tube. After removing the residual acid, the hydrolyzates were further analyzed to determine the monosaccharide composition using a model 1200 high-performance liquid chromatography (HPLC) apparatus (Agilent Technologies, Palo Alto, CA, USA), a Prevail Carbohydrate ES column-W250 × 4.6 mm 5 µm (Alltech, Nicholasville, KY, USA), and an ELSD 3300 detector (Alltech, Nicholasville, KY, USA) at a flow rate of 1.0 mL/min. The mobile phases (A and B) were acetonitrile and distilled water, respectively. The column was equilibrated in 75% mobile phase A as previously described [26] with the following modifications: 0–10 min linear gradient of 65% A, 10–20 min linear gradient of 50% A, 20–25 min of 50% A, 25–26 min linear gradient of 75% A, and 26–35 min of 75% A.

### 2.11. Nuclear Magnetic Resonance (NMR) Spectroscopy

^1^H and ^13^C NMR spectra were recorded with an Avance DRX600 spectrometer (Bruker, Beijing, China). The extracted polysaccharide were dissolved in deuterium (D_2_O, 99.9%) prior to the analysis, and exchangeable protons were substituted by deuterium after time lyophilization procedures [27]. Chemical shifts are shown in ppm.

### 2.12. Analysis of Sulfate-Group Content

The sulfate-group (–SO_3_H) content of polysaccharides was determined by the BaCl_2_-gelatin turbidity method [28,29]. In a typical procedure, 0.3% gelatin solution was prepared in hot water 70 °C and stored at 4 °C overnight. Two grams of BaCl_2_ was dissolved in gelatin solution and allowed to stand for 2–3 h at 25 °C. The preparation of standard solution of K_2_SO_4_: dried the K_2_SO_4_ powder under 105 °C, then accurately weighed 217.8 mg K_2_SO_4_ and dissolved in 200 mL 1 mol/L HCl. The preparation of polysaccharide solution: 8 mg extracted polysaccharide was hydrolyzed in 3 mL 1 mol/L HCl for 5 h at 105 °C in a sealed glass tube [28]. About 0.20 mL of K_2_SO_4_ stand solution (polysaccharide solution) was added to 3.8 mL of TCA and 1 mL of BaCl_2_-gelatin reagent, and the mixture was allowed to stand for 10–20 min. A blank was prepared with 0.2 mL of water instead of K_2_SO_4_ stand solution. The released barium sulfate suspension was measured at 360 nm by UV-visible spectrophotometer. The standard curve was shown in Figure S1. 

### 2.13. Statistical Analysis

Results were expressed as Mean ± SD from three independent experiments. Statistical analysis was performed using Design-Expert (Version 8.0.6 Trial) and SPSS (Version 20.0) statistical software. Data were analyzed using ANOVA followed by SNK-q test, a significant difference in values at *p* < 0.05.

## 3. Results and Discussion

### 3.1. Selection of Extraction Optimization Factors

**Figure 1:**
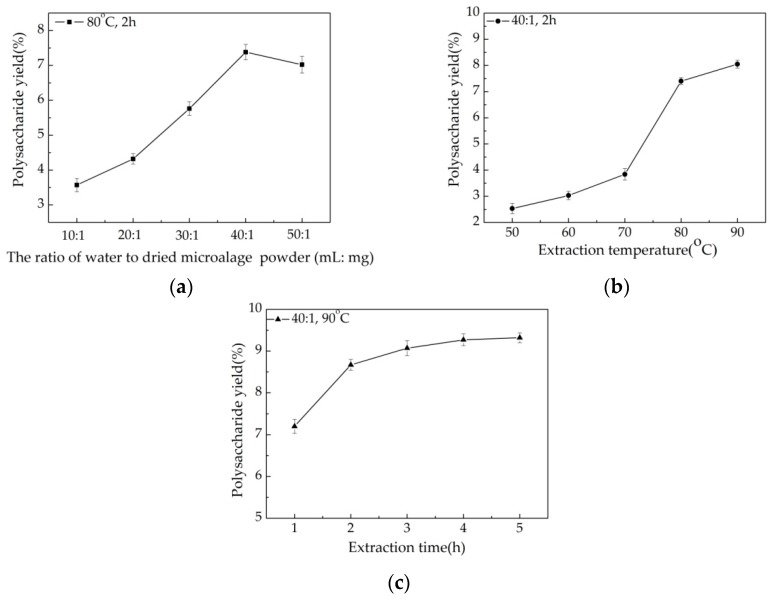
Effects of the ratio of water to dried algae powder (**a**) extraction temperature (**b**) and extraction time (**c**) on crude *Chlorella*-Arc polysaccharide extraction yield. Errors bars represent standard error for three replicates.

The effect of the water/powder ratio on the yield of crude polysaccharide is presented in Figure 1a. The extraction process was carried out at 10:1, 20:1, 30:1, 40:1, and 50:1, while the other two extraction factors were set as follows: extraction temperature 80 °C and extraction time 2 h. The crude polysaccharide yield increased from 3.57% to 7.38% with ratios increasing from 10:1 to 40:1. This might be explained by the fact that constituents can dissolve more effectively in more water, resulting in enhanced diffusivity of the solvent into cells, leading to an increased extraction yield [20,30,31]. A decrease in the extraction yield was observed with an increase in the water:powder ratio to 50:1. Therefore, we selected a ratio of water to dried microalgae powder of 40:1 as optimal.

As shown in Figure 1b, the extraction temperature was another factor that would influence the extraction yield. The extraction process was carried out at 50, 60, 70, 80, and 90 °C, other experimental conditions were as follows: the water/powder ratio 40: 1 and extraction time 2 h. The maximum extraction yield (8.05%) was obtained when the temperature was 90 °C. A considerable increase (from 3.84% to 7.40%) in the extraction yield was observed when the temperature increased from 70 to 80 °C. An increase in the extraction temperature resulted in the minimization of mass-transfer resistance [31]. Therefore, polysaccharides yields obtained at a higher temperature were significantly higher than those obtained at a lower temperature. Temperatures for the extraction of algae polysaccharides commonly range from 70 to 90 °C, but the extraction temperature has been extended above 90 °C [32]. High temperatures, however, may affect the yield and physiological activity of polysaccharides [3,30]. Based on these results, the temperature for extraction of polysaccharides in *Chlorella*-Arc was maintained below 90 °C.

The yield of crude polysaccharide extract increased as the extraction time was extended beyond 3 h (Figure 1c), when the water/powder ratio and extraction temperature were fixed at 40: 1 and 90 °C. The maximum extraction yield (9.32%) was observed at 5 h, but this time resulted in only a small increase in yield above that obtained at 3 h (9.07%). The extraction time is as important as the extraction temperature for polysaccharide yield [3,20]. From an industrialization perspective, an increase in time increases the cost of the extraction process. Therefore, 3 h was considered the optimal extraction time.

Based on this single-factor study, we adopted a water:powder ratio of 40:1, extraction temperature of 80 °C and extraction time of 3 h as optimal for the RSM experiments.

### 3.2. Box-Behnken Design (BBD) to Optimize Polysaccharide Extraction 

The response values for the 17 trials are shown in Table 2. The second-order polynomial model used can be characterized by the equation below (5).

(5) $$Y=8.42+0.77X_{1}+0.27X_{2}+0.49X_{3}+0.022X_{1}X_{2}+0.082X_{1}X_{3}-0.023X_{2}X_{3}-0.059X_{1}^{2}-1.73X_{2}^{2}-0.40X_{3}^{2}$$

Results of the ANOVA are shown in Table S1. A model *F*-value of 261.59 with a very low *p*-value (<0.0001) indicated that the model could be used to predict the crude polysaccharide extract yield [30,33]. In addition, the values of R-squared (0.9970), adj R-squared (0.9932), and pred R-squared (0.9647) revealed that the polynomial model was sufficiently accurate and generally available [3,34]. A low coefficient of variation (2.59%) clearly indicated significant precision and reliability of the experimental results [31].

### 3.3. Analysis of the Response Surface 

**Figure 2:**
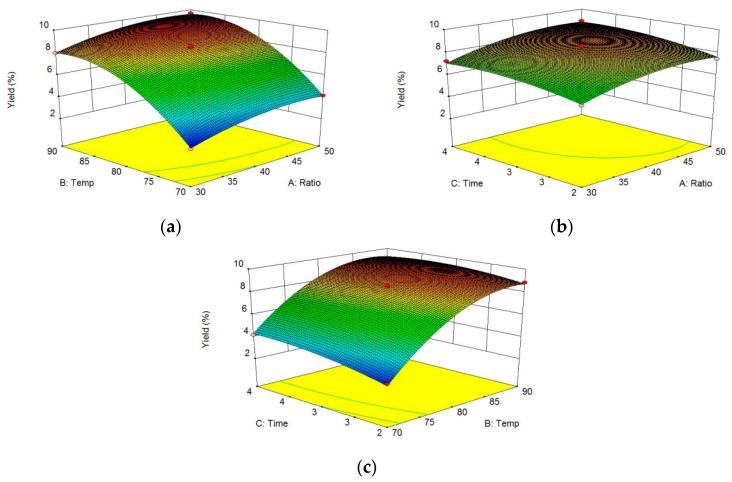
Response surface and contour plots for the effects of different parameters on *Chlorella*-Arc polysaccharide yields. (**a**) Ratio of water to dried microalgae powder and extraction temperature, with the extraction time held constant at 3 h. (**b**) Ratio of water to dried microalgae powder and extraction time, with the extraction temperature held at 80 °C. (**c**) Extraction temperature and extraction time, with the ratio of water to dried microalgae powder fixed at 40:1.

The effects of different factors on crude polysaccharide extraction were evaluated using three-dimensional (3D) graphs (Figure 2a–c). Two factors were depicted in the 3D response surface and two-dimensional (2D) contour plots, while another factor was held at zero. The shapes of the contour plots suggested the significance of the mutual interactions between the factors [31,34]. In the present study, the three factors each had a positive impact on the crude polysaccharide yield. Using Design-Expert, the optimum ratio of water to dried microalgae powder, extraction temperature, and extraction time to obtain crude polysaccharides were determined to be 47:1, 88 °C, and 3 h, respectively. The maximum predicted crude polysaccharide yield was 9.81%, and the experimental yield of crude polysaccharide was 9.62 ± 0.11%, (*n* = 3), which agreed with the predicted value. Shi et al. [32] obtained an polysaccharide extract yield of 4.48% from *Chlorella pyrenoidosa*. By optimizing the extraction of polysaccharides from this organism using the hot water extraction method, Hu et al. [4] obtained the highest reported polysaccharide yield of 7.78%. Tabarsa et al. reported a polysaccharide yield of 6.06% from *Chlorella vulgaris* following optimization of extraction conditions [13]. Qi et al. extracted *Chlorella ellipsoidea* polysaccharide and reported the yield was about 13.1%. The crude polysaccharide yield of our *Chlorella*-Arc approximately 9.62 ± 0.11% dry weight was obtained [14]. Therefore, *Chlorella*-Arc is a promising source of polysaccharides and its conspicuous polysaccharide yields offer an appreciable advantage over other *Chlorella* sp.

### 3.4. Purification of Crude Chorella-Arc Polysaccharide

**Figure 3:**
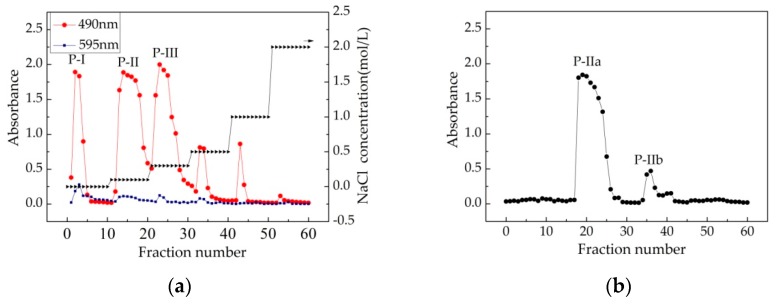

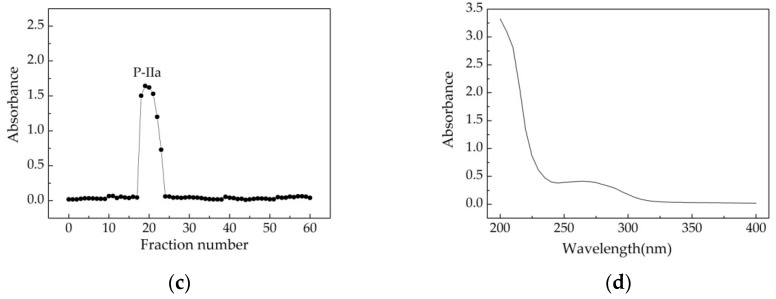
Isolation and physicochemical analysis of the polysaccharides extracted from *Chlorella*-Arc. (**a**) DEAE-Sepharose Fast Flow chromatogram elution curve of *Chlorella*-Arc polysaccharides. Fractions were collected and examined for metachromasia using phenol-H_2_SO_4_ (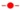) and Coomassie brilliant blue (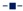). There were three major fractions in the crude *Chlorella*-Arc polysaccharide (P-I, P-II, and P-III). (**b**) Sephadex G-100 chromatogram elution profile of P-II; (**c**) Sephadex G-100 chromatogram elution profile of P-IIa; (**d**) UV-Vis spectrum of P-IIa

Following DEAE Sepharose Fast Flow chromatography purification, the crude polysaccharide from *Chlorella*-Arc was determined to contain three main fractions: P-I, P-II, and P-III (Figure 3a). P-II was further purified by Sephadex G-100 column chromatography and found to contain two peaks: P-IIa and P-IIb (Figure 3b). As shown in Figure 3c, P-IIa gave a single and symmetrical peak in the Sephadex G-100 column chromatograms, indicating that it represented a homogeneous and chromatographically pure polysaccharide [33]. The UV-Vis spectrum of P-IIa is shown in Figure 3d. The UV-Vis spectrum indicated that there were no optical absorption peaks at 260–280 nm. Therefore, P-IIa contains little proteins or nucleic acids, since proteins and nucleic acids absorb light at 260 and 280 nm, respectively. In addition, commasie brilliant blue staining (Figure 3a) confirmed low limit detection of proteins in the fraction. 

### 3.5. Antioxidant Properties 

Polysaccharides are abundant antioxidants. Microalgae polysaccharides have been widely reported as displaying antioxidant activities [4,24]. These previous reports lead us to check the antioxidant activities of the polysaccharides extracted from *Chlorella*-Arc. In order to evaluate the antioxidant activity of *Chlorella*-Arc polysaccharides, it was compared to the known standard antioxidant ascorbic acid.

#### 3.5.1. Scavenging Effects of Polysaccharide on DPPH Radical 

**Figure 4:**
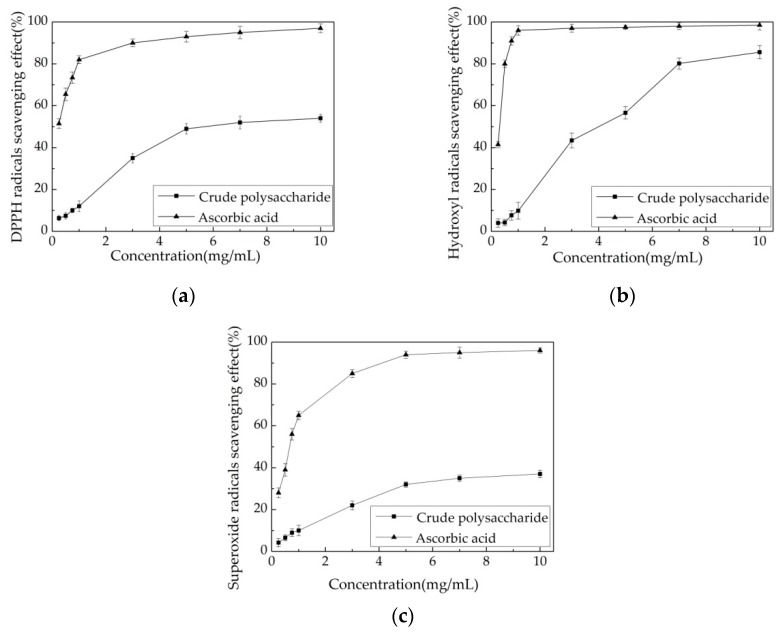
Antioxidant activities of *Chlorella*-Arc polysaccharides. (**a**) Scavenging effects on DPPH radical. (**b**) Scavenging effects on hydroxyl radical. (**c**) Scavenging effects on superoxide radical. Errors bars represent standard errors from three replicates.

DPPH radicals are stable free radicals that show a maximum absorption at 517 nm in methanol. The scavenging effect of antioxidants on DPPH radicals is a result of their proton-donating ability [35]. In DPPH radical tests, antioxidants can reduce the stable radical DPPH to yellow-colored diphenylpicryl hydrazine [20]. Based on this principle, the antioxidant activity of a substance can be measured by its ability to scavenge DPPH free radical. Figure 4a depicts the DPPH scavenging abilities of different concentrations of crude polysaccharide and ascorbic acid. The scavenging effect of the crude polysaccharide increased with increasing concentrations ranging from 0.25 to 10.0 mg/mL. The crude polysaccharide removed 49.10 ± 2.50% of DPPH radical at a concentration of 5.0 mg/mL. With higher concentrations, no further effect on DPPH radical was apparent. However, ascorbic acid displayed a stronger ability to scavenge DPPH radical. Specifically, ascorbic acid scavenged more DPPH radical (from 51.50 ± 2.30% to 82.00 ± 1.90%) as its concentration increased from 0.25 to 1.0 mg/mL. Hu et al. [4] reported the extraction of crude polysaccharide from *Chlorella pyrenoidosa* in an orthogonal experiment (L16(_4_^5^)). Almost all of the 16 extracts (10.0 mg/mL) showed high DPPH radical scavenging effects ranging from 29.67 ± 0.29% to 54.16 ± 4.49%. Chaiklahan et al. [3] extracted crude polysaccharide from *Spirulina* sp. at different temperatures (50, 70, 80, and 90 °C). At a concentration of 2.5 mg/mL, the DPPH radical scavenging effects ranged from 6.30 ± 2.10% to 31.00 ± 4.00%. Therefore, the crude polysaccharide from *Chlorella*-Arc obtained in this study removed DPPH free radicals similar to the polysaccharide from *Spirulina* sp. Ben Hafsa et al. reported water-soluble polysaccharides extracted from microalgae *Isochrysis galbana* (PEA) and *Nannochloropsis oculata* (PEB), showing a concentration dependent DPPH·radical scavenging activity. At concentration of 10 mg/mL, both PEA and PEB exhibit an antioxidant activity of 41.45 and 59.07% [36]. Custódio et al. indicated that organic extracts from *N. oculata* had also antioxidant properties with IC_50_ values between 4.93 and 7.31% [37]. Moreover, Balavigneswaran et al. reported that an ethanol soluble polysaccharidic extracts from *I. galbana* was active against DPPH (almost 40%) at 10 mg/mL [20].

#### 3.5.2. Scavenging Effects of Polysaccharide on Hydroxyl Radical 

Hydroxyl radicals in cells can easily cross cell membranes at specific sites, react with most biological macromolecules, and cause tissue damage and cell death. Thus, removing hydroxyl radicals is very important for the protection of living systems [38]. Presently, the hydroxyl radical scavenging effects of the crude extract were not as strong as ascorbic acid at any of the concentrations tested (Figure 4b). The scavenging effect of ascorbic acid on hydroxyl radical reached a plateau of 96.00 ± 2.30% at a concentration of 1.0 mg/mL. The ability of the crude polysaccharide to scavenge hydroxyl radical was concentration-dependent (Figure 4b). A significant increase in the scavenging activity of the tested crude polysaccharide was observed from 0.25–7.0 mg/mL. At a concentration of 7.0 mg/mL, the hydroxyl radical scavenging effect was 80.20 ± 2.70%. The crude polysaccharide displayed a slow increase in its ability to scavenge hydroxyl radical, with maximum activity of 85.60 ± 3.20% obtained at 10.0 mg/mL. Balavigneswaran et al. [20] extracted crude polysaccharide from *I. galbana* (20.0 mg/mL) and reported hydroxyl radical scavenging effects of approximately 60%. Xu et al. [39] reported a polysaccharide extracted from *Sphallerocarpus gracilis* with a hydroxyl radical scavenging effect of approximately 70% at 5.0 mg/mL. A chemical modification of polysaccharide is the form of a sulfated derivative produced a similar hydroxyl radical scavenging effect (80%) as the crude polysaccharide used in this study. Xia et al. reported polysaccharides extracted from marine diatom *Odontella aurita* was active against hydroxyl radical, at 10 mg/mL reaching the removal effect of 83.54 *±* 6.71% [40]. Zhao et al. purified different polysacchareide fractions of F1, F2, and F3 from *Laminaria japonica*, and found all the fractions exhibited obvious scavenging activity on the hydroxyl radical in a concentration-dependent manner, at 5 mg/mL, reaching 35%, 60%, and 65% scavenging efficiency [41]. Our results indicate that the crude polysaccharide from *Chlorella*-Arc has significantly higher antiradical activities than previously reported polysaccharide extracts at a dose of 7.0–10.0 mg/mL.

#### 3.5.3. Scavenging Effects of the Polysaccharide Extract on Superoxide Radical 

Superoxide radicals are oxidants present in most organisms and the source of free radical formed in vivo. Superoxide radicals and their derivatives damage DNA and cell membranes [42]. The effects of superoxide radical scavenging of the crude polysaccharide and ascorbic acid are shown in Figure 4c. The scavenging ability of ascorbic acid increased quickly at concentrations between 0.25 mg/mL and 5.0 mg/mL, whereas this increase slowed down at concentrations above 5.0 mg/mL. At a concentration of 5.0 mg/mL, the scavenging effect of ascorbic acid on superoxide radical was 94.00 ± 1.70%. In comparison, the crude polysaccharide showed less of a scavenging effect than ascorbic acid. At 5.0 mg/mL, the effect of the polysaccharide on superoxide radical was 32.10 ± 1.20%. Above 5.0 mg/mL, the increased concentration had no effect on superoxide radical. The extracted polysaccharide and a chemically modified sulfated derivative from *Sphallerocarpus gracilis* [39] showed superoxide radical scavenging effects of approximately 50% and 70%, respectively. Balavigneswaran et al. [20] extracted crude polysaccharide from *Isochrysis galbana* (5.0 mg/mL) and reported that the corresponding superoxide radical scavenging effect was about 42%. Moreover, Sun et al. isolated polysaccharides (IPSI-A, IPSI-B, and IPSII) from the marine microalgae *I. galbana* with the abilities of scavenging superoxide radicals of 33.8%, 32.3%, and 53.5% at 3.2 mg/mL [43]. The crude polysaccharide from *Chlorella*-Arc showed less activity in scavenging superoxide radical than some other polysaccharides reported and less of an effect on superoxide radical than on DPPH and hydroxyl free radical.

Based on these results, a concentration of 5.0 mg/mL was selected for further analysis. Table 3 shows the scavenging effects of *Chlorella*-Arc polysaccharides on DPPH, hydroxyl, and superoxide radical at concentrations of 5.0 mg/mL. Ascorbic acid showed higher antioxidant activities for these radical than *Chlorella*-Arc polysaccharides, and P-IIa showed greater scavenging effects than other *Chlorella*-Arc polysaccharides. The scavenging effects of P-IIa on DPPH, hydroxyl, and superoxide radical were 62.20 *±* 1.20%, 72.10 ± 1.50%, and 42.20 ± 1.60%. Shi et al. reported the biological activities of polysaccharide isolated from *Enteromorpha prolifera* (PE) are of insufficient potential for a range of purposes in the food and pharmaceutical industries. For instance, at 4 mg/mL the removal of DPPH, hydroxyl, and superoxide radical by PE reached 11.3%, 30.0% and 28.7% [44]. Chen et al. extracted *C. pyrenoidosa* polysaccharides (CPP) using different concentrations of ethanol for precipitation, and found CPP85 showed a great antioxidant activity at 2 mg/mL with the removal of DPPH, hydroxyl, and superoxide radical reaching 15%, 52%, and 55%. However, CPP70 exhibited stronger scavenging activity against superoxide, DPPH and hydroxyl radicals, when compared with CPP85 [24]. Many variables may affect the antioxidant activities of polysaccharides, including their molecular weight, monosaccharide composition, conformation, degree, and sulfate distribution pattern [23,45]. Therefore, a promising *Chlorella*-Arc polysaccharides and its conspicuous antioxidant activity deserved further investigation.

### 3.6. FTIR Spectroscopic Characterization 

**Figure 5:**
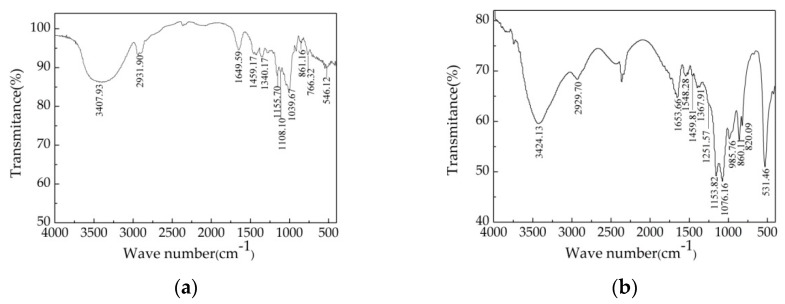
Fourier-Transform Infrared (FT-IR) spectra of Dextran T-500 (**a**) and P-IIa (**b**).

To obtain structural information, Dextran T-500 and P-IIa were analyzed by FTIR spectroscopy, which can be used to determine the structure of microalgae polysaccharides [25,46]. As shown in Figure 5, there were some overlapping peaks near 3400, 2931, 1650, 1459, 1155, and 860 cm^−1^ in the spectra of both *Chlorella*-Arc polysaccharides and the glucan standard (Dextran T-500). Different peaks (at 985, 820, and 531 cm^−1^) were observed in the fingerprint of P-IIa. A broad and intense peak around 3400 cm^−1^ (Dextran T-500: 3407.93 cm^−1^; P-IIa: 3424.13 cm^−1^) was attributed to the stretching vibration of O-H [47]. A weak peak at 2930 cm^−1^ (Dextran T-500: 2931.90 cm^−1^; P-IIa: 2929.70 cm^−1^) showed the stretching and bending vibration of C–H in the sugar ring. In addition, the peak at 1652 cm^−1^ (Dextran T-500: 1649.59 cm^−1^, P-IIa: 1653.66 cm^−1^) resulted from the stretching vibration in C=O and the carboxyl group [48]. The peak around 1455 cm^−1^ (Dextran T-500: 1459.17 cm^−1^; P-IIa: 1459.81 cm^−1^) represented CH_2_ bonding [49,50]. Two stretching peaks near 1155 and 1076 cm^−1^ were observed for the polysaccharide (Dextran T-500: 1155.70, 1108.10 cm^−1^; P-IIa: 1153.82, 1076.16 cm^−1^), which suggested the presence of the glycosidic linkages γ C–O–C and C–OH [51]. The peak at 1249.20–1262.80 cm^−1^ (P-IIa: 1251.57 cm^−1^) was due to the stretching vibration of S=O [29,52]. The characteristic peaks at approximately 860 and 820 cm^−1^ indicated both an α and β-anomeric configuration [53]. 

### 3.7. Monosaccharide Composition and Sulfate-Group Content Analysis 

**Figure 6:**
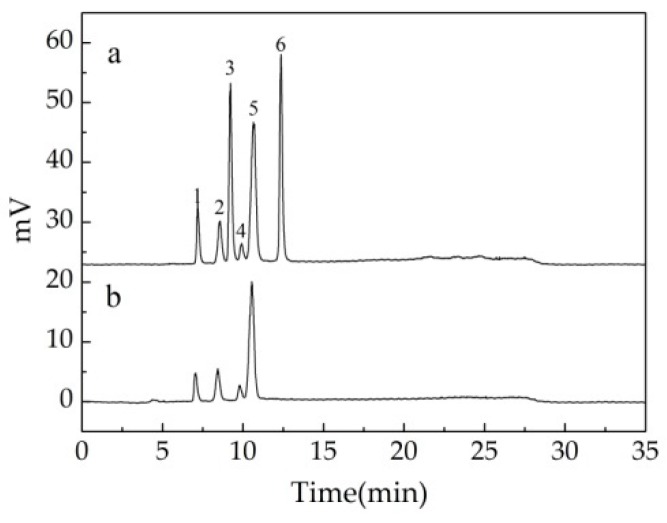
HPLC chromatogram of standard monosaccharides and hydrolyzed P-IIa. (**a**) Standard monosaccharides (1: rhamnose, 2: arabinose, 3: fructose, 4: glucose, 5: galactose, 6: sucrose). (**b**) HPLC chromatogram of hydrolyzed P-IIa.

The hydrolysate of P-IIa was further analyzed using HPLC to identify monosaccharides. As shown in Figure 6, the major monosaccharide of P-IIa was galactose (66.00%) followed by arabinose (16.32%), rhamnose (10.88%), and glucose (6.80%). The molar ratios of galactose:arabinose:rhamnose:glucose was 3.67:1.08:0.66:0.38. Hu et al. isolated a different polysaccharide from *C. pyrenoidosa*, of which galactose was a major monosaccharide with amounts of glucose, rhamnose, and mannose [54]. The polysaccharides of other *Chlorella* species, including *Chlorella vulgaris* [13] and *Chlorella ellipsoidea* [14], were both composed of glucose as the main monosaccharide, but other monosaccharides were varied. The chemical compositions of the microalgal polysaccharide were closely related to *Chlorella* species [14] and growing conditions, as well as the methods used for extraction. These findings provide a scientific basis for the use of polysaccharides from *Chlorella*-Arc. 

Sulfate-group content analysis showed the presence of sulfate in both crude polysaccharides (9.96 ± 0.25%) and P-IIa (11.58 ± 0.17%). These results were in agreement with previous researches [14] and confirmed the polysaccharide structure with sulfate ester groups (Figure 5b). The presence of –OSO_3_H in the polysaccharide could activate the hydrogen atom of the anomeric carbon. The higher activated capacity of the group, the hydrogen atom will have stronger donating capacity [39]. This might be one of the most important reasons that P-IIa acquiring a better antioxidant activity than crude polysaccharide (as shown in Table 3).

### 3.8. NMR Spectra Analysis

**Figure 7:**
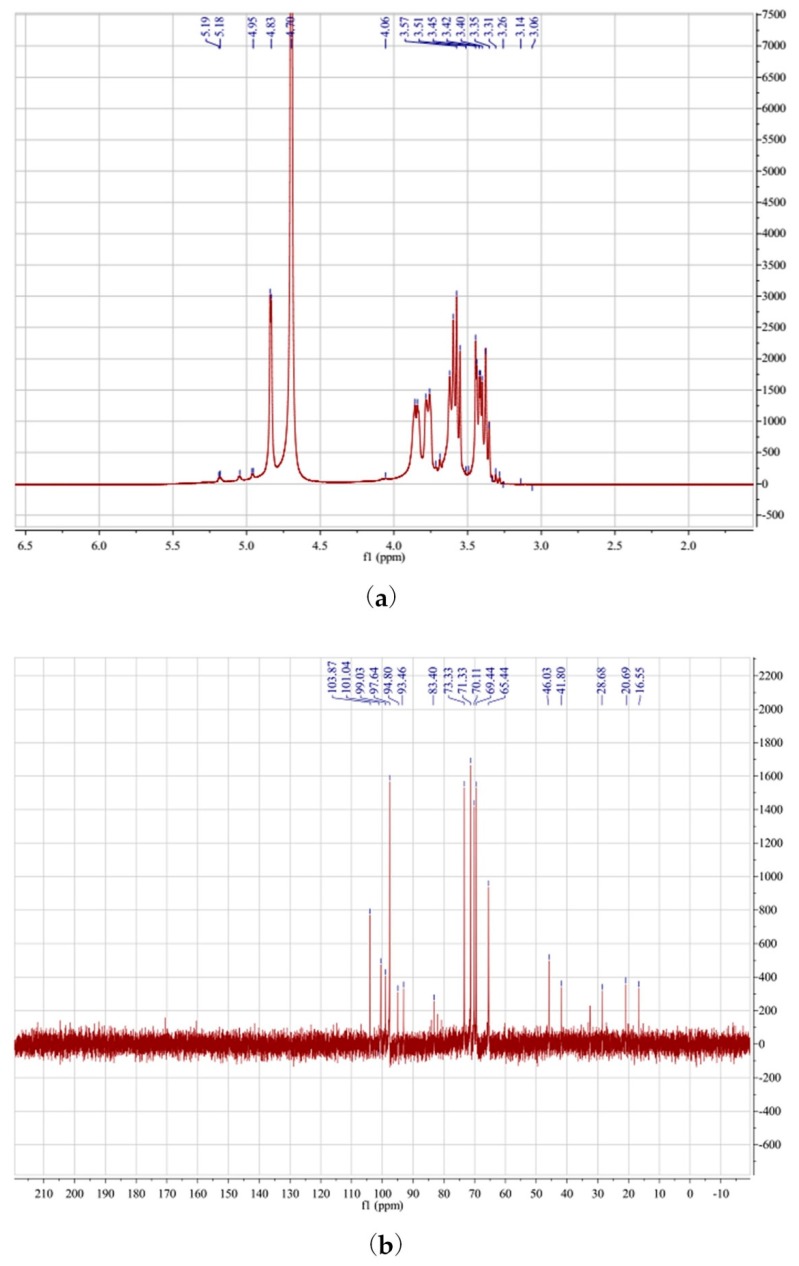
^1^H NMR spectrum (**a**) and ^13^C NMR spectrum (**b**) of P-IIa.

NMR spectroscopy was used to complete the structural characterization of P-IIa. Polysaccharides in the ^1^H NMR spectra (Figure 7a) typically show crowding in the range of δ3.0 to 5.0 ppm [53,55]. Chemical shifts from δ3.4 to 4.0 ppm were considered related to protons present at C-2–C-6. In the anomeric region of the ^1^H NMR spectrum of P-IIa, four main signals were apparent at δ5.19, 5.05, 4.95, and 4.83 ppm. These were both higher and lower than 5.0 ppm, indicating the presence of both α and β-configurations [53,56]. This agreed with the FT-IR spectrum analysis. The anomeric carbons characteristic of polysaccharides in the ^13^C NMR spectra show crowding from δ90 to 110 ppm [57]. The signals at δ95 to 101 ppm corresponded to α-anomeric carbons, and the signals at δ101 to 105 ppm corresponded to β-anomeric carbons in the ^13^C NMR spectrum [56], consistent with the ^1^H NMR spectrum. As shown in Figure 7b, the presence of C-1 signals (δ93.46–103.87 ppm) also indicated that the sugar residues were all in the pyran ring, as the resonance of the furan ring should be approximately δ107–109 ppm [55,57]. Based on the available data in the literature, the H-1 signal at δ5.19 ppm was likely due to ^1^H resonance of α-galactose residues, and the C-1 signal at δ99.03 ppm in the ^13^C NMR spectra supported this explanation. The H-1 signal at δ4.06 ppm and C-1 signal at δ101.43 ppm suggested that the polysaccharide contained β-arabinose residues [56], and the H-1 signal at δ4.83 ppm and C-1 signal at δ103.87 ppm indicated an β-anomeric configuration for (1*→*4)-linked β-d-glucopyranosyl units [22,55].

## 4. Conclusions

Extraction of crude polysaccharide from an arctic *Chlorella* strain, *Chlorella*-Arc, was optimized using RSM, purification, assessment of antioxidant activity, and preliminary structural characterization. Under the optimized conditions, *Chlorella*-Arc yielded a crude polysaccharide of approximately 9.62 ± 0.11% dry weight. After purification, three fractions (P-I, P-II, and P-III) were obtained. Antioxidant tests in vitro indicated greater antioxidant activity of P-IIa than other *Chlorella*-Arc polysaccharide fractions. The structural analysis revealed that P-IIa is a heteropolysaccharide with a pyran group and is composed mainly of rhamnose, arabinose, glucose, and galactose. These findings provide a scientific basis for the use of polysaccharides from *Chlorella*-Arc.

## Figures and Tables

**Table 1:** Experiment factors and levels for Box-Behnken design (BBD).

Factors	Code	Actual	Levels		
			−1	0	1
Ratio of water to dried microalgae powder (mL/g)	X_1_	x_1_	30	40	50
Extraction temperature (°C)	X_2_	x_2_	70	80	90
Extraction time (h)	X_3_	x_3_	2	3	4

**Table 2:** Box-Behnken Design (BBD) and the response values for extraction yields of crude polysaccharide.

Run	X_1_ (mL:g)	X_2_ (°C)	X_3_ (h)	Crude Polysaccharides Yield(%)	
				Actual	Predicted
1	40	80	3	8.35	8.42
2	30	80	4	7.25	7.07
3	30	70	3	2.59	2.66
4	30	90	3	7.99	8.01
5	40	80	3	8.50	8.42
6	40	80	3	8.61	8.42
7	40	80	3	8.34	8.42
8	50	90	3	9.65	9.58
9	40	70	4	4.20	4.31
10	50	80	4	8.86	8.76
11	50	70	3	4.16	4.14
12	50	80	2	7.45	7.63
13	40	70	2	3.04	2.87
14	40	80	3	8.30	8.42
15	40	90	4	9.07	9.24
16	40	90	2	8.84	8.73
17	30	80	2	6.17	6.17

**Table 3:** Antioxidant activity of *Chlorella*-Arc polysaccharides at 5.0 mg/mL.

Sample	Antioxidant Activity (%)		
	DPPH Radical	Hydroxyl Radical	Superoxide Radical
Crude polysaccharide	49.10 ± 2.50 ^c^	56.60 ± 2.50 ^d^	32.10 ± 1.20 ^c^
P-I	42.30 ± 1.70 ^b^	45.10 ± 2.10 ^b^	23.10 ± 1.30 ^b^
P-III	30.80 ± 1.20 ^a^	34.90 ± 1.20 ^a^	20.20 ± 1.50 ^a^
P-II	55.40 ± 1.30 ^d^	68.30 ± 2.40 ^e^	40.10 ± 1.40 ^d^
P-IIa	60.20 ± 1.20 ^e^	72.10 ± 1.50 ^f^	42.20 ± 1.60 ^d^
P-IIb	30.70 ± 1.10 ^a^	52.20 ± 1.60 ^c^	31.30 ± 1.20 ^c^
Ascorbic acid	93.00 ± 2.60 ^f^	97.50 ± 1.00 ^g^	94.00 ± 1.70 ^e^

In each column, different letters above the bars indicate a significant difference in values at *p* < 0.05 (ANOVA followed by SNK-q test).

## Supplementary Materials

The following are available online at http://www.mdpi.com/2073-4360/10/3/292/s1, Figure S1: Standard curve for determination of sulfate group content in microalgae polysaccharides by BaCl2- gelatin method. Regression equation is: Y = 0.31416x + 0.04545, R = 0.99647, Figure S2: UV-VIS spectrum of P-IIa, Table S1: ANOVA of the experimental results of the BBD.
