# Supplementary material for: Extraction Optimization, Purification, Antioxidant Activity, and Preliminary Structural Characterization of Crude Polysaccharide from an Arctic Chlorella sp
Source: Polymers (Basel). 2018 Mar 9;10(3):292. doi: 10.3390/polym10030292 (PMC6414885; doi:10.3390/polym10030292)
Supplement: Supplementary file 1 [file polymers-10-00292-s001.pdf]

Supplementary file

Article

Extraction Optimization, Purification, Antioxidant Activity,and Preliminary Structural Characterization of Crude Polysaccharide from an Arctic *Chlorella* Sp.

Hong Song, Meilin He, Chuankun Gu, Dong Wei, Yuqi Liang, Junmei Yan and Changhai Wang *

Jiangsu Provincial Key Laboratory of Marine Biology, College of Resources and Environmental Science, Nanjing Agricultural University, 1 Tongwei Road, Nanjing 210095, China; songhong8912@163.com (H.S.); hemeilin@njau.edu.cn (M.H.); 2016103007@njau.edu.cn (C.G.); 13260980201@163.com (D.W.); 18931330996@163.com (Y.L.); Ninglyq@163.com (J.Y.)

* Correspondence: chwang@njau.edu.cn; Tel.: +86-25-8439-6680

1. Standard Curve for Analysis of Sulfate Group Content

The sulfate-group (–SO_3H) content of polysaccharides was determined by the $BaCl_2$-gelatin turbidity method [28,29]. In a typical procedure, 0.3% gelatin solution was prepared in hot water 70 °C and stored at 4 °C overnight. Two grams of $BaCl_2$ was dissolved in gelatin solution and allowed to stand for 2–3 h at 25°C. The preparation of standard solution of K_2SO_4: dried the K_2SO_4 powder under 105 °C, then accurate weighed 217.8 mg K_2SO_4 and dissolved in 200 mL 1 mol/L HCl. The preparation of polysaccharide solution: 8mg extracted polysaccharide was hydrolyzed in 3 mL 1 mol/l HCl for 5 h at 105 °C in a sealed glass tube [28]. About 0.20 mL of K_2SO_4 stand solution (polysaccharide solution) was added to 3.8 mL of TCA and 1 mL of $BaCl_2$-gelatin reagent, and the mixture was allowed to stand for 10–20 min. A blank was prepared with 0.2 mL of water instead of K_2SO_4 stand solution. The released barium sulfate suspension was measured at 360 nm by UV-visible spectrophotometer. The standard curve was shown in Figure S1.

The standard curve was shown in Figure S1. The obtained regression equation is: Y = 0.31416x + 0.04545, R = 0.99647.

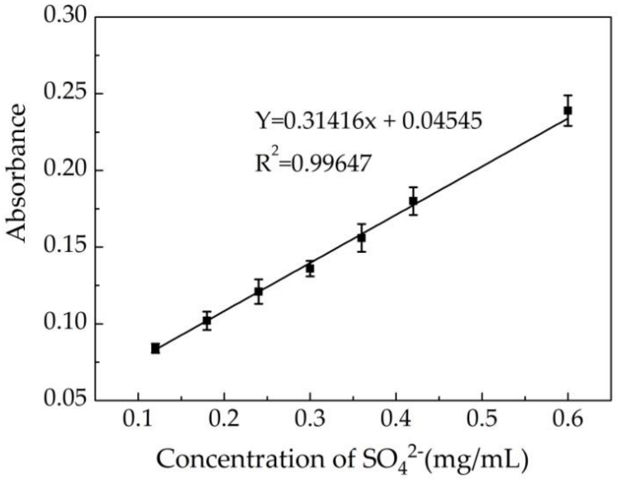

Figure S1. Standard curve for determination of sulfate group content in microalgae polysaccharides by

$BaCl_2$- gelatin method. Regression equation is: Y = 0.31416x + 0.04545, R = 0.99647.

2. **UV-VIS spectrum of P-IIa**

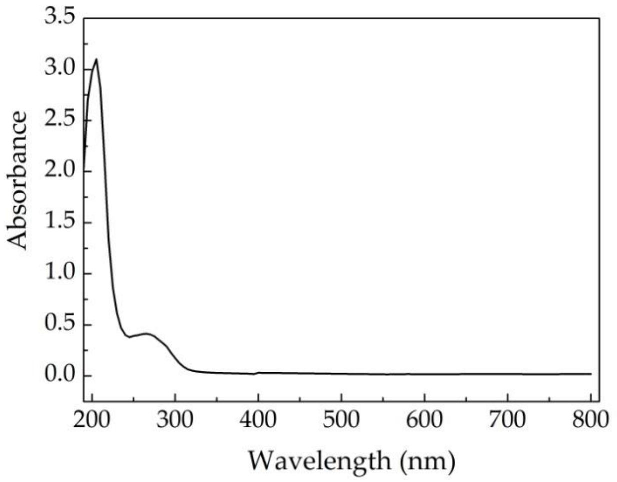

Figure S2. UV-VIS spectrum of P-IIa.

Table S1. ANOVA of the experimental results of the BBD.

Source	Sum of squares	df	Mean square	F-value	p-value prob >F
Model	80.70	9	8.97	261.59	< 0.0001[a]
X_1	4.68	1	4.68	136.58	< 0.0001[a]
X_2	58.10	1	50.81	1695.06	< 0.0001[a]
X_3	1.88	1	1.88	54.90	0.0001[a]
X_1X_2	2.02E-003	1	2.02E-003	0.059	0.8149
X_1X_3	0.027	1	0.027	0.79	0.4024
X_2X_3	0.22	1	0.22	6.31	0.0403[b]
X_1^2	1.46	1	1.46	42.58	0.0003[a]
X_2^2	12.66	1	12.66	369.22	< 0.0001[a]
X_3^2	0.67	1	0.67	19.53	0.0031[b]
Residual	0.24	7	0.034	-	-
Lack of fit	0.17	3	0.057	3.36	0.1363[ns]
Pure Error	0.068	4	0.017	-	-
Cor Total	80.94	16	-	-	-
Std. Dev.	0.19	-	-	-	-
C.V.%	2.59	-	-	-	-
Adeq-pre	48.733	-	-	-	-

R^2	0.9970	-	-	-	-
Adj R^2	0.9932	-	-	-	-
Pred R^2	0.9647	-	-	-	-

[ns] not significant; [a] significant at $p < 0.001$; [b] significant at $p < 0.05$
